# Anticoagulation Management and Monitoring during Pediatric Extracorporeal Life Support: A Review of Current Issues

**DOI:** 10.3389/fped.2016.00067

**Published:** 2016-06-22

**Authors:** Lindsay M. Ryerson, Laurence L. Lequier

**Affiliations:** ^1^Pediatric Critical Care, Stollery Children’s Hospital, Edmonton, AB, Canada

**Keywords:** pediatrics, anticoagulation, extracorporeal life support, heparin, bleeding

## Abstract

Anticoagulation is an imperfect science and is even more complicated in neonates and young children. The addition of the extracorporeal life support (ECLS) foreign circuit adds an additional layer of complexity. Anticoagulation goals during ECLS are to maintain a clot-free circuit and a hemostatically balanced patient. Unfractionated heparin (UFH) is the default gold standard anticoagulant as no large studies have been performed on any other anticoagulants. This review will focus on the advantages and disadvantages of the various methods to monitor UFH anticoagulation, discuss alternative anticoagulants, and examine bleeding and thrombotic complications during ECLS.

## Anticoagulation is an Imperfect Science and is More Complex in Neonates and Young Children

Unfractionated heparin (UFH) remains the anticoagulant of choice in pediatrics due to its short half-life and potential for reversal by protamine sulfate. Recommendations for laboratory monitoring of UFH in children have been extrapolated from adult recommendations (1, 2). The activated partial thromboplastin time (aPTT) and anti-Xa assays, both routine anticoagulation monitoring tests, were developed based on the adult hemostatic system. Infants and children have differences in hemostasis compared with adults. Procoagulant proteins (Factors II, VII, IX, X, XI, and XII) and inhibitors of coagulation [Protein C, Protein S, antithrombin (AT), and α2 macroglobulin] are present in different quantities, and the fibrinolytic system is relatively downregulated in young children. These normal physiologic differences are termed “developmental hemostasis” (3–5).

A prospective study examining the use of UFH and monitoring by standard tests (aPTT and anti-Xa) in neonatal and pediatric patients [not on extracorporeal life support (ECLS)] demonstrated poor correlation between UFH dose and both aPTT and anti-Xa (6). There was no correlation between aPTT and anti-Xa (6).

## Anticoagulation on ECLS Adds an Additional Layer of Complexity

The ECLS circuit contains a large foreign surface area that causes a massive inflammatory response, which affects both the cellular and plasma components of blood. Shear stresses imposed by the ECLS circuit activate cellular blood elements, including platelets and leukocytes, as well as plasma elements, such as von Willebrand factor. The initiation of ECLS is associated with a significant increase in activation markers of the coagulation system suggesting a consumptive coagulopathy (7). This may further exacerbate the loss of both pro- and anticoagulant proteins.

It is impossible to completely control the interaction between blood and the biomaterials of the ECLS circuit; this results in a shift to a hypercoagulable state. Consequently, antithrombotic therapy is necessary to prevent both circuit and patient thrombosis. Clinicians must balance the risk of bleeding complications with the need to maintain appropriate circuit flows and avoid thrombosis. A continuous UFH infusion is the default gold standard anticoagulant in ECLS; there are no large ECLS studies on any anticoagulants other than UFH. The optimal method to measure UFH efficacy on ECLS is unknown.

## Heparin is an Indirect Anticoagulant

Heparin is a heterogeneous mixture of branched glycoaminoglycans in saccharide chains. UFH is a mixture of different molecular weights (ranging from 3000 to 30,000 kDa) and corresponding saccharide chain lengths (1). Heparin is an indirect anticoagulant in that it requires AT for its primary anticoagulant action, inhibition of both thrombin and FXa. UFH does not inhibit clot-bound thrombin or thrombin bound to the ECLS circuit. The anticoagulant activity of UFH is variable for two reasons: (1) only one-third of an administered dose of UFH has the specific, active pentasaccharide sequence that binds AT and (2) the anticoagulant profile of UFH is influenced by the chain length of the molecules (1).

Unfractionated heparin binds to AT *via* a unique glucosamine unit that contains a specific, active pentasaccharide sequence (1). The UFH/AT complex is responsible for the anticoagulant action of UFH by inhibition of Factors IIa (thrombin), Factors Xa, IXa, Xia, and XIIa. Once bound, the UFH/AT complex has 1000× the inhibitory effect compared with AT alone. The remaining two-third of the UFH dose, without the active pentasaccharide sequence, has minimal anticoagulant action at therapeutic doses and may, in fact, inhibit anticoagulation by binding to plasma proteins and activating platelets (1).

Heparin molecules are made up of different lengths of saccharide chains. UFH inhibits thrombin by AT binding *via* the high affinity pentasaccharide sequence as well as binding directly to the coagulation enzyme (1). Inhibition of FXa requires only the binding of AT to UFH *via* the specific pentasaccharide sequence. Thrombin (IIA) and Factor Xa are the most sensitive to inhibition by UFH/AT complex (1). However, only heparin molecules of 18 or greater saccharide units are able to catalyze the interaction of both AT and thrombin. If the heparin saccharide chain is too short, it is unable to catalyze thrombin inhibition (1). As a result, there may be inhibition of FXa, but not of thrombin, which leads to variable anticoagulation. This heterogeneity makes monitoring of anticoagulation difficult.

## Standard Monitoring of Coagulation is Imperfect

Monitoring of coagulation is done *in vitro*, which eliminates the effects of the endothelium *in vivo*. This flaw is a major limitation in our understanding of how coagulation occurs in the patient. A second limitation is that the majority of our coagulation tests are plasma-based tests, partial functional measures of coagulation, and not whole blood tests that incorporate platelet function and assessment of clot strength. Table 1 summarizes the advantages and disadvantages of different UFH monitoring techniques.

**Table 1:** **UFH monitoring techniques**.

Test	Advantages	Disadvantages
ACT	• Inexpensive • POC • Whole blood test	• Measures end point of the clotting cascade, but does not solely tell you about UFH effect
aPTT	• Accepted means of titrating anticoagulation therapy for both UFH and DTI • POC now available	• High degree of intra- and interpatient variability especially in infants • Less reliable in critical illness
Anti-Xa	• Specific measure of UFH effect based on the ability of UFH to catalyze AT’s inhibition of factor Xa • Better association with UFH dose	• Elevated plasma-free hemoglobin and hyperbilirubinemia will underestimate UFH activity by anti-Xa
TEG/ROTEM	• POC • Whole blood test • Provides information about both clot strength and fibrinolysis	• Limited availability

*UFH, unfractionated heparin; POC, point-of-care; AT, antithrombin; DTI, direct thrombin inhibitor; TEG, thromboelastography; ROTEM, rotational thromboelastometry*.

### Activated Clotting Time

The activated clotting time (ACT) is the only routine whole blood test performed on ECLS. ACT measures the time in seconds of whole blood to form fibrin clot after the addition of various coagulation activators. ACT does not measure clot strength. ACT was invented in the 1960s for cardiopulmonary bypass (CPB) and since then has been applied to various settings requiring UFH anticoagulation. ACT results will vary based on many factors including platelet number and function, fibrinogen level, coagulation factor deficiencies, patient temperature, hemodilution, and technical factors (8). Hence, it does not represent, solely, UFH effect. Furthermore, different ACT machines yield different results either because of different coagulation activators or because they are measuring different end points. For example, a common point-of-care (POC) ACT in the United States is the Hemochron System, either the Hemochron Response or the Hemochron Signature Elite. Both of these systems look at the time of whole blood to form clot, but measure this end point differently. The Hemochron Response System uses magnet technology; clot endpoint occurs with a specific displacement of the magnet. The Hemochron Signature Elite System detects clot formation by LED optical detectors that measure the speed of the blood sample. The instrument recognizes that a clot endpoint has been achieved when the movement decreases below a predetermined rate. Unfortunately, these systems cannot be used interchangeably (9).

The I-STAT ACT is another POC ACT system. It is similar to traditional ACT tests except that the endpoint is indicated by the conversion of a thrombin product other than fibrinogen, and an electrochemical sensor is used to indicate this conversion. There is variability between different ACT monitoring systems, which makes comparisons between centers using different systems impossible (10).

Baird and colleagues retrospectively reviewed over 600 pediatric ECMO patients and found only a modest correlation (*r* = 0.48) between ACT and UFH dose suggesting that ACT is not an accurate tool for monitoring UFH anticoagulation during pediatric ECMO (8). Other reports examining the correlation of UFH and ACT on ECLS provided mixed results varying from either no statistical correlation (11, 12) to a better correlation within individual patients (13).

### Activated Partial Thromboplastin Time

Historically, dose monitoring of patients receiving UFH has been with the aPTT. The aPTT is a plasma-based test that measures the time from Factor XII activation to fibrin formation, after addition of calcium and a reagent. A prospective, adult study suggested that an aPTT between 1.5 and 2.5× normal was associated with a decreased risk of recurrent venous thromboembolism (2). Based on this adult study, the therapeutic range for the aPTT was set at 1.5–2.5× the patient’s pre-therapy baseline aPTT; however, this was validated neither in randomized controlled trials nor in children.

The use of aPTT for monitoring UFH is based on the assumption that the patients’ baseline aPTT is comparable to normal controls; this assumption is critical for the linear relationship between UFH dose and aPTT. Unfortunately, the baseline aPTT in sick pediatric patients is very different from normal controls, which limits the utility of aPTT as a measure of UFH effect. Increased levels of non-specific acute phase reactants, Factor VIII, and fibrinogen often escalate in critically ill infants and children; increased levels of these proteins may falsely shorten the aPTT masking true UFH effect (6). As a result, aPTT demonstrates a high degree of intra- and interpatient variability, which resulted in increased number of blood tests for monitoring and frequent dose changes. Consequently, many clinical laboratories and clinicians have substituted the anti-Xa assay for UFH monitoring.

### Anti-Xa Assay

The anti-Xa assay (alternatively called the heparin level) is a measure of UFH effect based on the ability of UFH to catalyze AT’s inhibition of Factor Xa. Anti-Xa is a plasma-based test that does not incorporate platelet function. The anti-Xa assay evaluates only one chemical reaction of the UFH–AT complex; it does not measure inhibition of thrombin. The anti-Xa is used as a surrogate measure of the overall anticoagulant activity of UFH. The same adult study that set the therapeutic aPTT at 1.5–2.5× the patient’s baseline aPTT demonstrated that this therapeutic aPTT range corresponded to an anti-Xa activity of 0.3–0.7 U/mL (2).

A number of single-center ECLS case series have found an improved association of anti-Xa with UFH dose as well as an associated decrease in blood sampling, decrease in blood product transfusion, and less complications, both thrombotic and hemorrhagic, while using anti-Xa as a part of UFH monitoring protocol during ECLS (13–17). The anti-Xa assay is a chromogenic assay, which can limit its utility in ECLS as the anti-Xa is falsely decreased when there is elevated plasma-free hemoglobin or bilirubin (18).

Newall and colleagues prospectively compared UFH concentration *via* protamine titration and UFH effect *via* aPTT, anti-Xa, and thrombin clotting time (TCT) in a group of pediatric patients in the cardiac catheterization laboratory (19). Protamine titration is considered the gold standard of UFH concentration in plasma. Their results refute the pediatric recommendations for UFH management extrapolated from adult trials. Agreement between all measures of UFH effect and protamine titration was poor (19). They propose that the current recommendations for management of UFH in pediatrics are inappropriate, which demands either different tests for monitoring UFH efficacy or alternative anticoagulants.

### Viscoelastic Testing

Viscoelastic tests [thromboelastography (TEG) and rotational thromboelastometry (ROTEM)] examine whole blood clot formation (coagulation) and dissolution (fibrinolysis). They are limited by static conditions (no flow) as well as the exclusion of vascular endothelium, but they are the only option available to evaluate clot strength. Additional benefits include POC testing that can be performed at the bedside and at the patient’s temperature. Additional information from viscoelastic testing includes time until initial fibrin formation, the kinetics of fibrin formation and clot development, the ultimate strength and stability of the fibrin clot, clot lysis, and platelet function (20). TEG and ROTEM are not yet widely available; only 43% of centers use viscoelastic tests as part of their routine anticoagulation monitoring on ECLS (21). There are limited reports documenting improved outcomes when TEG was incorporated into a bundled anticoagulation protocol (22).

## Antithrombin Replacement

The optimal AT level for any patient receiving UFH anticoagulation is unknown. In infants and children with escalating UFH requirements and/or clinically inadequate anticoagulant effect, relative AT deficiency may be a contributing factor. There is insufficient experience to provide evidence-based recommendations for AT concentrate use in children on ECLS.

If low AT levels are confirmed, AT replacement may be considered (23–26). AT concentrate and recombinant AT are now available and some centers will prophylactically replace it for AT activity <50–80% (<0.5–0.8 U/mL). Some centers will treat low AT levels only if there is evidence of reduced UFH effect based on low anti-Xa levels or minimal UFH effect on TEG kaolin versus TEG heparinase samples.

Of the ECLS programs that use AT replacement routinely as part of their anticoagulation protocol, most target levels from >50 to >100%. The use of AT concentrate is preferred over the administration of frozen plasma (FP) as giving standard bolus infusions of FP does not easily achieve adequate AT levels (may need >20 mL/kg for FP to obtain clinical AT effect as standard plasma contains only 1 U/mL for AT).

The UFH infusion can be decreased (up to 50%) prior to AT replacement because of the potential for significant augmentation of UFH’s anticoagulant effect. ACT and anti-Xa should be checked 30–60 min after AT administration to evaluate UFH effect.

## Alternative Anticoagulants

It is impossible to provide high quality evidence supporting the use of UFH alternatives on ECLS. However, there is increasing experience with alternative anticoagulants.

### Direct Thrombin Inhibitors

Direct thrombin inhibitors (DTIs) are not dependent on AT for their anticoagulant effect. They act directly to inhibit both circulating and clot-bound thrombin. DTIs are superior to UFH in their ability to inhibit clot-bound thrombin (27). Bivalirudin is a potentially attractive DTI as it is cleared mainly by intravascular proteolytic degradation and less so by the kidneys (27). Bivalirudin may be used as the initial anticoagulant on ECLS or patients may be transitioned to bivalirudin in cases of heparin-induced thrombocytopenia (HIT) or heparin resistance. Argatroban and lepirudin are other possible DTIs, but they rely on hepatic and renal clearance, respectively, which are organ systems often compromised on ECLS. Bivalirudin has a short half-life of 25–35 min, which is even shorter in children (27). Bleeding from bivalirudin is potentially reversible with activated Factor VII. Bivalirudin is approved for use as an anticoagulant in adult patients undergoing angioplasty. There is off-label experience using bivalirudin infusions in adult patients with HIT demonstrating successful anticoagulation with a target aPTT of 1.5–2.5× normal (28).

There is minimal experience with bivalirudin in pediatric populations and even less in pediatric ECLS. Nagle and colleagues report a series of 12 children transitioned to bivalirudin because of UFH resistance, UFH failure, or HIT on ECLS (29). Their maintenance dose range of bivalirudin was very wide due to significant interpatient variability. They followed aPTT, but do not report their target range. Retrospective adult studies comparing UFH and bivalirudin on ECLS demonstrated no difference in thromboembolic complications (30, 31).

There are evolving challenges with use of bivalirudin on ECLS. Bivalirudin does not work well when there is blood stasis, which is common on ECLS requiring the change of different circuit components and is worrisome for the development of intraventicular clot if the heart is not ejecting (32). Bivalirudin also falsely elevates INR (30). ACT does not appear to be useful in monitoring of bivalirudin, and while aPTT may be limited by poor linearity and reproducibility, it is the most common monitoring test. Most centers will initially target a lower therapeutic range (aPTT of 1.5–2.0× normal) and increase the target aPTT to 1.5–2.5× normal as necessary for optimal circuit mechanics. Table 2 compares UFH and bivalirudin.

**Table 2:** **Comparison of UFH and bivalirudin**.

Anticoagulant	Mechanism of action	Dosing range	Reversible	Monitoring tests	Limitations
UFH	Indirect – needs AT for maximal therapeutic effect	10–70 U/kg/h	Protamine sulfate	ACT, aPTT, anti-Xa, and TEG/ROTEM	• Only one-third of UFH molecules has pentasaccharide sequence that binds AT, which leads to variable anticoagulation • Does not inhibit clot-bound thrombin • Thrombocytopenia
Bivalirudin	Direct thrombin inhibitor	0.3–1.2 mg/kg/h	No specific antidote, but bleeding may be mitigated with factor VIIa	aPTT	• Expect INR will increase • Thrombosis with blood stasis (intracardiac and circuit)

*UFH, unfractionated heparin; AT, antithrombin; TEG, thromboelastography; ROTEM, rotational thromboelastometry*.

### New Oral Anticoagulants

Rivaroxaban is a highly specific inhibitor of Factor Xa, which prevents the formation of the thrombin burst by inhibiting coagulation at the point of amplification (33). There is limited *in vivo* evidence in pediatrics. Oral factor-IIa inhibitors, such as dabigatran, are in use in adult patient for the prevention and treatment of thromboembolism. There is no data in adult ECLS. Their enteral administration and the paucity of pediatric studies will limit their use in pediatric ECLS.

### Factor XIIa Inhibitors

A new and alternative anticoagulation therapy based on an antibody to Factor XIIa was studied in an animal extracorporeal circuit model (34). This model demonstrated that a recombinant human FXIIa neutralizing antibody prevented fibrin deposition and thrombosis in the extracorporeal circuit and did not increase clinical bleeding.

## Prevention and Management of Bleeding and Thrombotic Complications

### What Is Considered Significant Bleeding on ECLS?

Both bleeding and thrombotic complications are associated with increased morbidity and mortality (35, 36). Complications reported in the ECLS literature are based on Extracorporeal Life Support Organization (ELSO) registry data that are non-specific. A patient hemorrhagic complication is defined as one that requires transfusion or other intervention. We suggest that clinically relevant bleeding be defined more specifically. Major bleeding may be defined as clinically overt bleeding associated with a hemoglobin drop of at least 20 g/L in a 24-h period; bleeding that is retroperitoneal, pulmonary, or involves the central nervous system (CNS); bleeding >20 mL/kg over 24 h; or bleeding that requires surgical intervention (37). Minor bleeding may be defined as bleeding of 10–20 mL/kg over 24 h.

If there is excessive bleeding, particularly in postcardiotomy patients, holding the UFH for 4–6 h is reasonable. In some circumstances where bleeding is difficult to control, the UFH may be held up to 12 h or longer until bleeding is controlled.

### Antifibrinolytic Therapy

There are no standardized dosing regimens for antifibrinolytic therapies on ECLS. These therapies are most commonly required to manage postoperative bleeding in the ECLS patient. Some centers use aminocaproic acid (Amicar) (38) or tranexamic acid (Cyklokapron) prophylactically in postcardiotomy patients, some use only prior to surgical intervention or when managing excessive bleeding, while others do not use at all. Amicar may be loaded (100 mg/kg IV bolus over 1 h) and then followed by continuous infusion of 25–33 mg/kg/h IV for at least 48 h. Tranexamic acid typically run as an infusion from 10 to 16 mg/kg/h IV until bleeding subsides.

### Activated Factor VII

There is conflicting data as to the safety and efficacy of activated Factor VII to manage refractory bleeding on ECLS (39–41). Activated Factor VIIa is considered for patients with severe, refractory hemorrhage only after the optimization of coagulation factors, fibrinogen, and platelets. Factor VIIa is usually given with platelets at a dose of 30–90 mcg/kg IV every 8 h for 3 doses.

### Thrombotic Complications in the Patient and the Circuit

Circuit thrombosis becomes clinically relevant if it requires circuit intervention or is associated with severe hemolysis. Patient thromboses, particularly CNS infarcts, are likely underreported as not all patients receive neuroimaging after ECLS decannulation. Clinically relevant patient thrombosis, defined as an intracranial infarct demonstrated by neuroimaging after a change in a patient’s neurologic exam or screening test, may be the most practical definition of a patient thrombotic complication. Other patient thrombotic complications, such as renal or splenic infarcts, require imaging for diagnosis.

### Heparin-Induced Thrombocytopenia

Heparin-induced thrombocytopenia is an antibody-mediated event that results in paradoxical arterial and venous thrombosis. Since thrombocytopenia is common in critically ill patients and on ECLS, HIT is frequently suspected, but rarely diagnosed in pediatrics. The initial ELISA-based immunoassay that tests for the presence of antibodies against heparin-platelet factor 4 (PF4) conjugate is sensitive, but not specific (42). A serotonin release assay is typically performed as confirmatory testing when the immunoassay is positive (42).

### Optimal Blood Product Replacement

There is a lack of studies that guide transfusion practices in ECLS patients. ELSO recommends that the patient is transfused to maintain a hemoglobin level of 140–150 g/L (43). A recent survey by Bembea and colleagues found variable thresholds for blood product transfusion (21). The majority of pediatric centers used a hematocrit of 35% with a range of 25–40% as a trigger for transfusion of red blood cells (RBCs) (21). The median platelet count that triggered platelet transfusion was 100,000 × 10^9^/L with a range of 50,000–200,000 × 10^9^/L (21).

Given the association of positive fluid balance and mortality in pediatric critical care as well as the concern about storage issues with RBC, some centers are re-examining their transfusion triggers. Fiser and colleagues recently examined the relationship between the transfusion of RBCs and changes in mixed venous saturation (SvO2) and cerebral regional tissue oxygenation, as measured by near infrared spectroscopy in patients supported with ECLS (44). The median hematocrit was 0.37. The majority of transfusions resulted in no significant change in either SvO2 or cerebral NIRS. There was no relationship between pre-transfusion hematocrit and the change in either SvO2 or cerebral NIRS suggesting that RBC transfusion did not significantly alter global tissue oxygenation (44).

Agerstrand and colleagues performed a retrospective study of 38 adults receiving ECLS for ARDS after initiation of a blood conservation protocol that included a transfusion trigger of hemoglobin <70 g/L, use of low dose anticoagulation targeting an aPTT of 40–60 s, and autotransfusion of circuit blood during decannulation (45). Only 24 patients (63%) required transfusion over a median ECLS run of 9 days (45). Twenty-eight patients (73.7%) survived to hospital discharge, which is comparable to the published literature. High survival rates with a hemoglobin transfusion trigger of 70 g/L suggest that a conservative transfusion strategy may be applicable to this population (45).

## Conclusion

Unfractionated heparin anticoagulation on ECLS is extremely variable due to the heterogeneity of UFH molecules and UFH’s inability to inhibit clot-bound thrombin. As a result, it can be difficult to have a hemostatically balanced patient and a clean circuit. In addition, the ECLS patient population is critically ill, which may limit the use of standard anticoagulation monitoring tests. These tests are limited in that they are only partial functional measures of anticoagulation. Global measures of anticoagulation that use whole blood, incorporate platelets, and examine clot strength are becoming more widely available.

Bleeding and thrombotic complications are common in pediatric ECLS and are associated with increased mortality. Bivalirudin, a direct thrombin inhibitor, is a promising alternative anticoagulant as it avoids many of the limitations of UFH, but prospective rigorous studies are urgently needed. There remains a concern about increased risk of intracardiac thrombosis with bivalirudin if the heart is not ejecting. Additional studies are required to challenge the current dogma and evaluate triggers for blood product replacement.

Interested readers should refer to the ELSO Anticoagulation Guidelines and Patient-Specific Protocols available on the ELSO website (http://ELSO.org) for further reading.

## Author Contributions

All authors listed, have made substantial, direct and intellectual contribution to the work, and approved it for publication.

## Conflict of Interest Statement

The authors declare that the research was conducted in the absence of any commercial or financial relationships that could be construed as a potential conflict of interest.
